# From Nature to Lab: A Review of Secondary Metabolite Biosynthetic Pathways, Environmental Influences, and In Vitro Approaches

**DOI:** 10.3390/metabo13080895

**Published:** 2023-07-28

**Authors:** Zubair Altaf Reshi, Waquar Ahmad, Alexander S. Lukatkin, Saad Bin Javed

**Affiliations:** 1 Plant Biotechnology Laboratory, Department of Botany, Aligarh Muslim University, Aligarh 202002, India; zubairreshi26@gmail.com (Z.A.R.); waquar9721@gmail.com (W.A.); 2 Department of General Biology and Ecology, N.P. Ogarev Mordovia State University, 430005 Saransk, Russia

**Keywords:** plant tissue culture, environmental stress, defence action, industrial use, sustainable production

## Abstract

Secondary metabolites are gaining an increasing importance in various industries, such as pharmaceuticals, dyes, and food, as is the need for reliable and efficient methods of procuring these compounds. To develop sustainable and cost-effective approaches, a comprehensive understanding of the biosynthetic pathways and the factors influencing secondary metabolite production is essential. These compounds are a unique type of natural product which recognizes the oxidative damage caused by stresses, thereby activating the defence mechanism in plants. Various methods have been developed to enhance the production of secondary metabolites in plants. The elicitor-induced in vitro culture technique is considered an efficient tool for studying and improving the production of secondary metabolites in plants. In the present review, we have documented various biosynthetic pathways and the role of secondary metabolites under diverse environmental stresses. Furthermore, a practical strategy for obtaining consistent and abundant secondary metabolite production via various elicitation agents used in culturing techniques is also mentioned. By elucidating the intricate interplay of regulatory factors, this review paves the way for future advancements in sustainable and efficient production methods for high-value secondary metabolites.

## 1. Introduction

Plant metabolites, both primary and secondary, play crucial roles in the growth and survival of plant species [1]. Primary metabolites, such as lipids, proteins, carbohydrates, amino acids, and vitamins, directly contribute to essential cellular processes like cell division, respiration, and photosynthesis, crucial for plant growth and development [2]. In contrast, secondary metabolites have multifunctional roles, primarily involved in defence and interactions with the environment [3]. Additionally, they contribute to plant colour, specific fragrances, flavours, and responses to various stresses. The concept of plant secondary metabolites in plant biology was introduced by Kossel et al. [4]. Secondary metabolites are highly reactive, and their accumulation is influenced by both biotic and abiotic stress conditions, which can have detrimental effects on physiological and morphological characteristics like leaf number, leaf area, plant height, and productivity [1].

Secondary metabolites play a crucial role in helping plants cope with different stress conditions. The response is initiated through the activation of plant defence mechanisms, triggered by the recognition of foreign agents through sensors and receptors in plants [5]. Moreover, plant survival and productivity rely on the expression of defensive transcriptional factors [3]. Detection of threat signals and increased production of secondary metabolites through elicitation contribute to the downstream expression of transcriptional factors [3]. For example, in response to abiotic stress, the expression of β-lycopene cyclase in *Bixa orellana* and phytoene synthase in *Daucus carota* is elevated, leading to the accumulation of carotenoids [5,6]. The production of secondary metabolites is highly dependent on the developmental stages and physiological conditions of the plant. In various interactions, such as mutualism observed in root nodules of legumes, or antagonistic relationships, such as pathogenicity and herbivory, secondary metabolites play a pivotal role by exerting irreversible effects [7].

Teoh [8] classified plant secondary metabolites into various groups based on functional groups and chemical structure. These groups include terpenes (including volatile compounds, sterols, and carotenoids), polysaccharides, phenolic compounds, phytoalexins (sulfur-containing compounds), alkaloids (nitrogen-containing compounds), flavonoids, and hydrocarbons. Almost all of these metabolites contribute significantly to defence against stressful situations. Plant hormones, such as abscisic acid (ABA), jasmonates (JA), polyamines, and salicylic acid (SA), are also involved in responding to environmental stresses, and their accumulation often results from various biotic and abiotic stresses and the response to elicitors and other signalling molecules [9].

The low-molecular-weight secondary metabolites have garnered significant interest among researchers due to their dramatic implications for pharmaceutical, nutritional, and industrial purposes. Recent advancements in the research of secondary metabolites have focused on finding a reliable source for production and extractions of important secondary metabolites for industrial use [10].

In vitro culture-based elicitation mechanisms are considered advantageous for the production of secondary metabolites as they offer independence from environmental conditions and reduced the risk of microbial contamination. In vitro micropropagation techniques are efficient for mass-producing secondary metabolites applicable to various industrial and pharmaceutical companies. Other natural products derived from plants, including steroids, codeine, morphine, pilocarpine, digitoxin, and quinine, are used in various pharmaceutical products [8].

Environmental and developmental factors influence the synthesis and accumulation of secondary metabolites in plants greatly. Therefore, this review aims to provide a comprehensive summary of how various environmental conditions influence the synthesis and accumulation of secondary metabolites. The qualitative and quantitative aspects of the environment can serve as tools to improve the accumulation of secondary metabolites in plants by modifying their growing conditions as well as the use of in vitro culture techniques for sustainable production and extraction of these secondary metabolites for industrial use.

## 2. Biosynthesis of Secondary Metabolites in Plants

Secondary metabolites in plants are categorized into distinct chemical groups based on their biosynthetic pathways: phenolic compounds, terpenes and steroids, and nitrogenous compounds. These chemical structures determine the function and stress adaptation of secondary metabolites. Various stresses such as drought, pathogenesis, herbicides, salinity, and heavy metals promote the accumulation of secondary metabolites [11].

Plants develop numerous adaptive strategies to overcome harsh conditions by upregulating the synthesis and accumulation of secondary metabolites. The production levels of these metabolites are greatly influenced by factors such as growing temperature and environmental constraints [12]. Under suitable conditions, more than 100,000 secondary metabolites are synthesized through various metabolic pathways. The interrelation between the synthesis of primary and secondary metabolites is fundamental for most plants.

**Figure 1:**
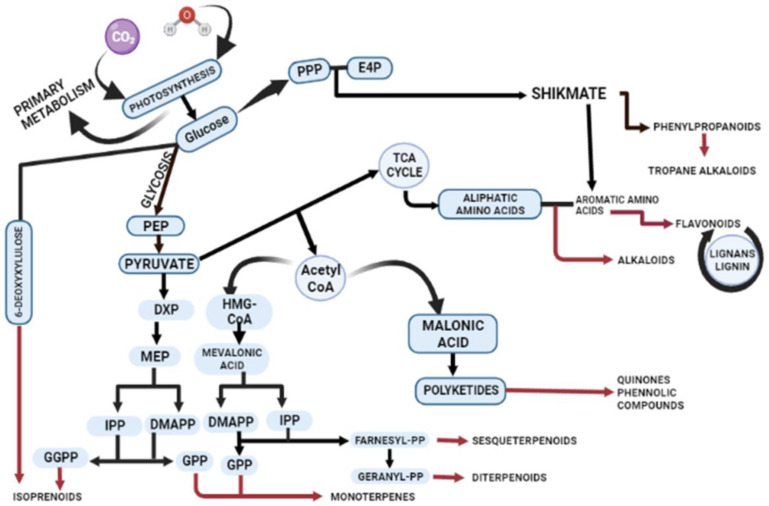
Schematic illustration of biosynthetic pathways for secondary metabolite production. The figure demonstrates the intricate biosynthesis of secondary metabolites and their interconnections with primary metabolism within plants. Plant cells employ diverse mechanisms through major pathways including mevalonic acid (MVA) and the 2-C-methylerythritol 4-phosphate (MEP) and shikmate pathway to synthesize terpenes, phenols, flavonoids, and alkaloids. Abbreviations: phosphoenolpyruvate (PEP); 1-deoxy-d-xylulose 5-phosphate (DXP); 4-hydroxy-3-methylbut-2-enyl diphosphate (HMBPP); isopentenyl pyrophosphate (IPP); dimethylallyl pyrophosphate (DMAPP); geranyl pyrophosphate (GPP); 2-C-methyl-l-erythritol 4-phosphate (MEP); 1-deoxy-D-xylulose 5-phosphate (DXP); acetyl coenzyme A (Acetyl-CoA); β-hydroxy β-methylglutaryl-CoA (HMG-CoA); geranylgeranyl diphosphate (GGPP); erythrose 4-phosphate (E4P); and pentose phosphate pathway (PPP).

Primary and secondary metabolites are distinct in their distribution, chemical structure, and functional roles in plants. Figure 1 illustrates the production of secondary metabolites and their interconnections with primary metabolism within the plant cell. The alternative mechanisms involved in the biosynthesis of secondary metabolites lead to common products, such as phenols, flavonoids, and terpenes (Figure 1). However, the critical precursors for secondary metabolites are primary metabolites. The shikimic acid pathway and Krebs cycle produce essential precursors required for the production of phenolic metabolites [13]. The aromatic molecules synthesized by the shikimic acid pathway play featured roles in electron transport, antioxidants, wound response, structural agents, and defence systems [14]. The aromatic amino acids L-tryptophan (L-Trp), L-tyrosine (L-Tyr), and L-phenylalanine (L-Phe) serve as precursors for secondary metabolite synthesis produced by the shikimate pathway [15]. Chorismate is the final product in the seven-step pathway of shikimic acid, and it serves as the starting material for the biosynthesis of secondary metabolites. Chorismate mutase, aminodeoxychorismate synthase, and isochorismate synthase play regulatory roles in higher plants for chorismate, which is also the precursor of folate, phenylalanine, phylloquinone, and tryptophan [16]. In fungi, the AROM complex undergoes catalysis by enzymes, such as 3-dehydroquinate dehydratase (DHD) and shikimate dehydrogenase (SDH), which facilitate the third and fourth reactions within the shikimate pathway. In plants, SDH and DHD act bifunctionally, while they have single functions in *Escherichia coli*.

The synthesis of terpenes involves two other pathways: the MEP (2-C-methylerythritol 4-phosphate) and MVA (mevalonic acid) pathways, occurring in plastids and the cytosol, respectively. The MEP pathway is responsible for synthesizing isoprene, plastoquinone, terpenes, and phytol [17], while the MVA pathway is involved in the synthesis of triterpenes, sterols, and sesquiterpenes [18].

In the MVA pathway, mevalonate is formed from acetoacetyl-CoA through the enzymatic activity of 3-hydroxy-3-methylglutaryl-CoA reductase (HMGCR) [19,20]. Subsequently, mevalonate undergoes phosphorylation and decarboxylation to yield sterol and nonsterol isoprenoids. Phosphomevalonate kinase (PMK) catalyzes the phosphorylation of mevalonate, resulting in the formation of mevalonate-5-pyrophosphate [21,22]. The final step involves an ATP-dependent decarboxylation reaction, wherein mevalonate-5-pyrophosphate is converted to IPP by the enzymatic action of phosphomevalonate decarboxylase [23]. Terpenes are synthesized in distinct compartments of the cell through the action of terpene synthases [24]. Fungi and bacteria execute the malonic acid pathway for the synthesis of phenolic compounds [24]. Essential products of glycolysis, such as glyceraldehyde-3-phosphate and pyruvate, are required for the production of dimethylallyl diphosphate (DMADP) and pyrophosphate (IPP). DMADP and IPP serve as universal building blocks for all isoprenoids in numerous cellular compartments [18].

Amino acids such as lysine, tyrosine, and tryptophan act as precursors for nitrogenous secondary metabolites. Enzymes like chalcone synthase (CHS) and phenylalanine ammonia-lyase (PAL) regulate the phenolic content under different stress conditions. For example, plants respond to salt stress and UV rays by producing alkaloids through the regulation of hyoscyamine 6β-hydroxylase and tryptophan decarboxylase, respectively. Flavonoids are specifically synthesized through the phenylpropanoid pathway, where phenylalanine is transformed into 4-coumaroyl-CoA, which enters the biosynthetic pathway. Various biosynthetic enzymes, including reductases, hydroxylases, and Fe(II)/2OG-dependent oxygenases, catalyze oxidative transformations, leading to different flavonoid subclasses [25]. Flavonoids all originate from chalcone scaffolds produced by chalcone synthase, and transferases modify the flavonoid backbone, altering the physiological activity of the resultant flavonoid [26]. The phenylpropanoid pathway also plays a significant role in activating the antioxidant system in plants by eliciting phenolic compounds like vanillic acid, caffeic acid, cinnamyl acid, and gallic acid [27].

The biosynthesis of secondary metabolites in plants involves intricate pathways, connecting both primary and secondary metabolism. These compounds play crucial roles in stress adaptation and various physiological functions, making them essential for plant survival and defence.

## 3. Secondary Metabolites as Regulators of Growth and Development

Secondary metabolites, such as glucosinolates and flavonoids, play crucial roles in regulating the growth and development of plants, as well as responding to various environmental conditions.

Glucosinolates are involved in different mechanisms that modulate plant growth and development. For example, aliphatic 3-hydroxypropylglucosinolate inhibits meristematic root growth in *Arabidopsis* and other plant species through the rapamycin pathway [28,29]. However, the exact mechanism by which 3-hydroxypropylglucosinolate functions is yet to be fully understood. Aliphatic glucosinolates in *Vicia faba* and *Arabidopsis* regulate stomatal closure through a peroxidase-mediated receptor kinase [30]. Additionally, the enhanced expression of the myrosinase gene TGG1 in guard cells plays an active role in stomatal regulation through various signalling pathways [31]. Indole-3-carbinol, an immediate product of indole glucosinolate, negatively affects *Arabidopsis* root growth when applied after exogenous wounding. This effect is due to the binding of indole-3-carbinol at an allosteric enzyme site, mediating the regulation between auxin and its receptor TIR1 [32].

Flavonoids, a diverse group of phenolic compounds, also have significant regulatory roles in plant growth and development under different environmental conditions. Changes in the flavonoid biosynthetic pathways influence various physiological, growth, and developmental processes [33]. A study by Maloney et al. [34] demonstrated the effect of flavonols on the developmental stages of pollen in tomatoes. The study compared two mutant tomato varieties with a defective *F3H* gene and a wild-type variety. The mutation in the *F3H* gene resulted in decreased flavonoid accumulation in plants. This reduction in flavonoid content was critically associated with impaired pollen tube growth and germination, as well as compromised pollen tube integrity. Flavonoids also play a role in reducing reactive oxygen species (ROS) levels and affecting developmental processes in guard cells, leaves, and roots [35,36]. Exogenous application of flavonoids has been shown to influence auxin transport [37]. In vitro studies on *Arabidopsis* demonstrated that flavonoids act as potential inhibitors of auxin transport [38]. The study of the transparent testa (tt4) chalcone synthase mutant of *Arabidopsis*, compared to the wild-type, showed phenotypic differences such as decreased plant height, reduced root growth, and altered auxin transport due to the presence of naringenin, a precursor of flavonoid biosynthesis [38].

## 4. Secondary Metabolite Production in Plants in Response to Different Environmental Factors

**Figure 2:**
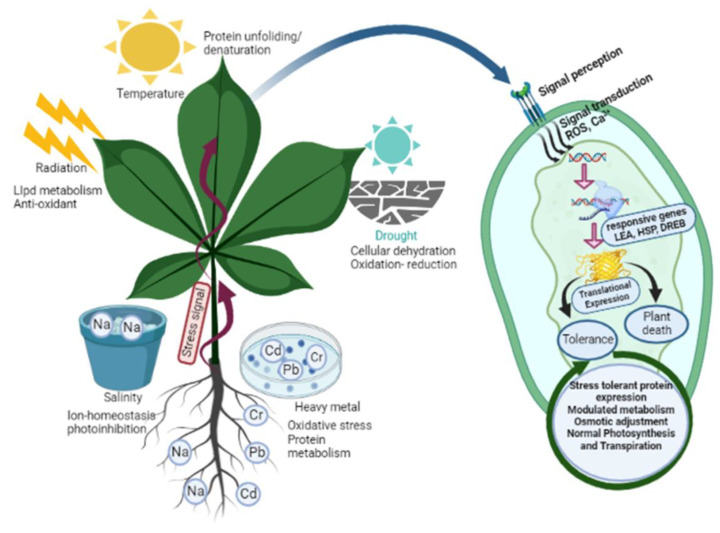
The schematic presentation illustrates the diverse array of abiotic stresses, including drought, salinity, temperature, heavy metals, and radiation impacting plant growth and development. In response to these challenges, plants have evolved an array of remarkable adaptation mechanisms. These include the expression of stress-tolerant proteins, finely modulated metabolism, osmotic adjustment, stomatal closure, synthesis of vital secondary metabolites, activation of specific antioxidants, and meticulous maintenance of ionic balance. These strategies help plants confront and overcome environmental hardships. Abbreviations: late embryogenesis abundant (LEA); heat shock proteins(HSP); dehydration-responsive element binding (DREB); reactive oxygen species (ROS).

Plants, being sessile organisms, face potential threats from environmental or pathogenic stressors. These stresses can lead to osmotic imbalances, physiological and biochemical changes, and cellular dehydration, ultimately resulting in the death of the affected plant (Figure 2). To defend themselves, plants have evolved three different response mechanisms. Ephemeral desert plants respond by avoiding stress by regulating their life cycle, as the fragile plants lack effective mechanisms to survive stress, while resistant plants respond with efficient defensive mechanisms to counter various stresses. This defensive system is modulated by plants through alterations and modifications in membrane structure, cell cycle and division remodelling, changes in photosynthetic activity, conductance, and transpiration rates, which collectively affect growth, metabolic activity, and the physiology of metabolic compounds [39] (Figure 2). Signalling processes prompt primary metabolism, allowing for the accumulation of enhanced secondary metabolites that initiate a defensive mechanism against various environmental constraints [40] (Table 1). These secondary metabolites protect the plant against both biotic and abiotic stress conditions. Environmental stresses such as salinity, water availability, radiation, chemical exposure, and temperature fluctuations significantly impact the content of secondary metabolites. For instance, exposure to UV irradiation, herbicide treatment, and other environmental stresses often enhances the accumulation of phenylpropanoids [41]. The levels of magnesium, potassium, and iron can influence the increased accumulation of phenolic compounds in roots [41]. The antioxidant activity of polyamines and phenyl amides in tobacco and beans highlights the crucial role of secondary metabolites in coping with environmental constraints [42].

### 4.1. Salt Stress

Salt stress exerts a negative impact on plant growth and productivity [64]. Plants respond to salt stress by undergoing changes in their metabolic activity to counteract the toxicity induced by excessive salt levels [64]. Salinity often causes osmotic imbalance and ionic toxicity in plants, leading to membrane disruption and cellular dehydration in the cytoplasm [65]. These changes in ionic and osmotic components can result in either an increase or decrease in the accumulation of plant secondary metabolites. In vitro studies have revealed a decline in anthocyanin accumulation in salt-sensitive species [66], while the decrease in phenolic, anthocyanin, and flavonoid content under salt stress conditions was less pronounced in salt-tolerant clones [67]. Proline, which regulates stress signals and serves as an osmotic buffer, energy sink, and protector of membranes and proteins, shows increased accumulation in the roots of Alfalfa under salt stress [68]. Various studies have observed the accumulation of polyphenols and flavonoids under salinity stress in plants like *Hordeum vulgare* and *Cakile maritma* [69,70]. Transcription factors GmCAM4 and GmERFo57 play crucial roles in providing resistance to salinity stress in *Glycine max* [71]. *Spinacia oleracea* demonstrates resistance against saline soil by enhancing the production of 20-Hydroxyecdysone (20E) [72]. Furthermore, alkaloids such as vincristine and reserpine in *Catharanthus roseus* and *Rauvolfia tetraphylla*, respectively, increase under saline conditions [73].

### 4.2. Drought Stress

Drought severely impacts plants’ biochemical and physiological processes, causing disruptions in the electron transport chain and leading to the production of reactive oxygen species (ROS) like H_2_O_2_, OH^−^, and O_2_^−^. These ROS inflict oxidative damage on lipids, nucleic acids, and proteins, and also disrupt the photosynthetic mechanism of plants. Drought severity varies among plant species and is often accompanied by increased solar radiation and temperature [74]. The stress disturbs plant metabolism, reduces cell turgidity and signalling, and impairs energy storage, plasma membrane structure, and resource allocation [75].

To cope with drought stress, plants have developed response mechanisms that involve the accumulation of secondary metabolites such as phenolics, terpenes, and alkaloids. These secondary metabolites help regulate ionic balance and enzyme activity, repair oxidative damage, and maintain the connection between the phenotype and genotype of plants [76,77]. However, increased production of these metabolites can lead to reduced biomass in some plant species [78]. For instance, under drought stress, plants like *Artemisia annua* and *Catharanthus roseus* increase secondary metabolite production by several times [79]. Drought stress induces elevated expression of *PAL* genes, responsible for flavonoid synthesis, in roots of *Scutellaria baicalensis* [80]. In *Chenopodium quinoa*, the accumulation of saponin changes under different drought stress conditions [81]. Drought stress also affects the carotenoid and chlorophyll ratio in plants. Notably, *Bellis perennis* and *Ophiorrhiza mungos* show enhanced production of camptothecin, alkaloids, and phenolic compounds, respectively, under drought conditions [82,83].

Drought stress has varying effects on the concentrations of secondary metabolites in the roots and leaves of *Bupleurum chinensis* plants. For example, saikosaponin concentrations in the roots increase during vegetative and reproductive growth, while leaf rutin concentrations decrease significantly during these stages [84].

These studies underscore the intricate interplay between abiotic stresses and plant physiological responses, leading to diverse responses of secondary metabolites against drought stress.

### 4.3. Temperature Stress

Elevated temperatures lead to reduced development and early leaf senescence in plants. The impact of temperature on the production of secondary metabolites varies among different plant species [85,86]. Heat treatments can cause enzyme inactivation, and lipid and protein denaturation negatively affect membrane integrity. Under temperature stress, carotenoid and β-carotene accumulation in *Brassicaceae* are reduced [85]. Acidic pH combined with a high temperature in hairy root culture promotes the accumulation of flavonolignans in *Silybum marianum* [87]. Some plants, like *Melastoma malabathricum*, show increased anthocyanin and biomass production at lower temperatures compared to higher temperatures [88]. *Daucus carota* responds to heat shock by increasing terpene accumulation, while the production levels of α-terpinolene decrease at the same temperature. Short-term temperature treatment enhances isoprene production synthesized through the nonmevalonate pathway (MEP), which is believed to counteract the negative effects of heat shock [89,90]. Different temperature ranges can significantly affect the level of secondary metabolites in plants, as observed in *Amaranthus cruentus* [91]. Salicylic acid and phenolic content in flowering plants [92] and phenolic compounds in gymnosperms [93] were found to decrease with increasing temperature. In tea plants, the concentration of catechin levels increases when treated with higher temperatures [94].

### 4.4. Light, UV, and Ionization Radiation

Light plays a critical role in plant metabolism and the production of secondary metabolites. Stable light intensity regulates photosynthesis and dry matter accumulation. However, abnormal irradiation can lead to photodamage and negatively affect photosynthetic reaction centres, causing photoinhibition, which limits plant survival, growth, and development [95]. The production and accumulation of secondary metabolites from various precursor elements depend on light intensity and the lengths of the photoperiod [96,97]. Different plant species respond differently to the quality, intensity, and length of photoperiod (day length) [98]. Appropriate light intensity regulates the accumulation and quality of flavonoids, alkaloids, spermine, and hexadecanoic acid [97,99]. For example, *Melastoma malabathricum* cell cultures exposed to different light intensities and full irradiance or complete darkness showed varying anthocyanin yield and biomass accumulation [88]. In vitro cultured seedlings of *Hyptid marrubiodes* exhibited increased production of flavonoids in red light, while rutin accumulation increased in white and blue light [100]. UV light treatment generally has a positive effect on the synthesis of secondary metabolites; however, at higher irradiation doses of UV-C and UV-B, some plants may react with decreased growth, abnormal metabolic activity, and photosynthesis [101].

Exposure of *Asparagus officinalis* to UV-B results in higher activity of peroxidase and phenylalanine ammonia-lyase, which ultimately enhances the accumulation of quercetin-4′-O-monoglucoside [102]. In *Catharanthus roseus*, UV-B light exposure significantly influences the production of vinblastine and vincristine, both used in treating leukemia and lymphoma [103]. Moreover, a combination of UV light (280–320 nm) with red light stimulates anthocyanin production in *Malus domestica* [104], while UV irradiation on *Fagopyrum esculentum* increases the content of quercetin [105]. Several studies have shown that UV light treatment is associated with increased phenolic compounds and ROS scavenging systems [106].

Additionally, the duration of light exposure plays a role in affecting secondary metabolite concentration. For example, light intensity and wavelength had a considerable effect on the accumulation of secondary metabolites in the leaves of *Flourensia cernua* [107]. Similarly, *Ipomea batatus* demonstrated differential effects on the concentration of phenolic compounds and flavonoids with enhanced levels observed upon exposure to longer light duration [108]. In green algae, *Dunaliella baradawil*, the accumulation of indoleamines like melatonin and serotonin increased under different photoperiod treatments [109].

### 4.5. Heavy Metal Stress

Plants respond differently to metal toxicity, resulting in variations in the accumulation and production of secondary metabolites due to the differential mobility of heavy metals that alter plant components, leading to reduced biosynthesis of plant defence compounds [110]. The adaptation and tolerance of plants against heavy metal stress are associated with the signalling molecules responsible for the synthesis and accumulation of secondary metabolites [111]. For example, *Hypericum perforatum* showed decreased accumulation of hypericin and pseudohypericin when exposed to nickel stress [112]. Nickel can either increase or inhibit the synthesis and accumulation of anthocyanin content in different plant species [113,114]. *Brassica juncea* treated with Fe, Mn, and Cr showed increased plant oil content [115]. Toxicity under high copper chloride (CuCl_2_) conditions activates the defence mechanism in *Viburnum ichangense*, leading to the accumulation and biosynthesis of ichangoside and phenolic diglycoside [116]. *Salix purpurea* exhibits an increased accumulation of phenolic compounds and salicylic acid under Cu- and Ni-stress conditions [117].

## 5. Other Factors Influencing Secondary Metabolism in Plants

In addition to the stresses mentioned earlier, plants encounter various other stress conditions, and to cope with these challenges, they have developed improved mechanisms of avoidance, biological detoxification, accumulation, and exclusion [12]. Each plant genotype responds uniquely to different abiotic stress factors, necessitating the study of specific plant species considering their individual stress responses rather than a single stress condition. In response to these stresses, plants produce specific secondary metabolites to mitigate the negative effects induced by environmental factors [96].

Gaseous toxins significantly impact the production of plant secondary metabolites. For instance, gaseous pollutants like sulfur dioxide (SO_2_) can damage photosynthetic activity by entering plants through stomatal openings during the photosynthetic process, triggering defence mechanisms in plants [118]. Treating *Brassica oleracea* with sodium hydrosulfide at a lower concentration (0.5 and 1 mM) leads to an enhanced content of sinigrin, carotenoids, phenolic compounds, and anthocyanins [119].

Pesticides represent another potential abiotic stress factor that affects plant metabolism and biosynthetic pathways. However, their specific impact on the production of secondary metabolites is not fully understood. Some studies have shown that herbicides and fungicides can influence levels of flavonoids, tropane alkaloids, and anthocyanins [120,121]. Many plants have developed special mechanisms to degrade and neutralize the hazardous effects of pesticides [122].

Recent research has highlighted the positive role of mineral nutrients in biomass production and the biosynthesis of secondary metabolites. Mineral nutrition can either increase or decrease plant growth and the accumulation of secondary metabolites, depending on the genotype, developmental stage, and environmental conditions of the studied plant [123,124,125]. Deficiencies in phosphorus, an essential element in primary metabolism involving energy currency molecules like ATP and ADP, can lead to reduced development and increased production of anthocyanin compounds [126]. For instance, *Satureja hortensis* exhibited increased accumulation of rosmarinic acid, known for its antioxidant, antiviral, and anti-inflammatory activities, when supplemented with nitrogen fertilizer [127]. However, excessive nitrogen application negatively influenced secondary metabolite synthesis in *Labisia pumila* due to reduced phenylalanine ammonia-lyase (PAL) activity and low photosynthetic rates [128]. Copper can inhibit deaminase oxidase activity, which is crucial in the biosynthetic pathways of secondary metabolites like cadaverine and putrescine [129,130]. Other mineral nutrients such as sulfur, potassium, and phosphate also impact the synthetic pathways of phenolic compounds and phenylpropanoids in various plant species [123,124,125]. For example, potassium treatment enhances the synthesis of phenolic compounds in the leaves of *Ocimum basilicum* [131].

## 6. Defence Action through Secondary Metabolism in Plants

Plants face a wide range of environmental stresses such as temperature fluctuations, irradiation, heavy metals, salinity, and gaseous toxins [132]. In response to these abiotic stresses, plants accumulate and synthesize various secondary metabolites, with different species producing distinct compositions [133]. The production of secondary metabolites serves as a defence mechanism against abiotic stress and plays a crucial role in regulating the growth and productivity of plants [96]. When exposed to stress, plants induce gene networks involved in the regulation and production of cellular molecules like detoxifying enzymes and osmoprotectants, which act as the first line of defence [134,135]. Reactive oxygen species (ROS), known for their damaging effects, are rapidly generated upon sensing stress signals in plants [136]. ROS, acting as secondary messengers, stimulate defence genes and modulate protein structure [137,138]. They also impact signalling through respiratory burst oxidase homologue D (RBOHD) and disrupt cell-to-cell communication. ROS signalling activates stress-related genes in Arabidopsis by activating various transcription factors [30]. In salt-tolerant citrus plants, the accumulation of oxidized and S-nitrosylated proteins is enhanced by priming the antioxidant activity of H_2_O_2_ under NaCl stress, whereas control plants exhibit greater susceptibility to stress [139].

**Figure 3:**
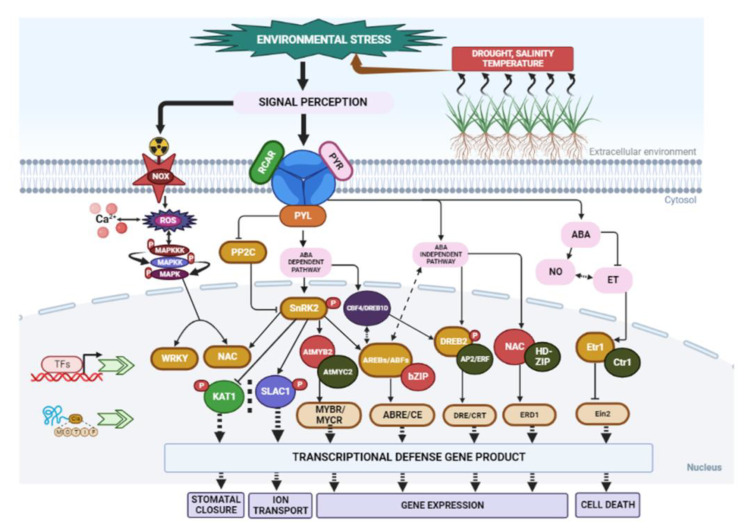
Schematic overview of signal perception, transduction, and transcriptional regulatory networks under environmental stresses, including drought, heat, and other stresses. In plants with and without ABA, abiotic stress activates the ABA signalling system. In the absence of ABA, PP2C phosphatases (negative regulators) interact with SnRK2 kinases (positive regulators) and inhibit their activation. Signal transduction is blocked due to the inactivation of SnRK2s. The presence of ABA permits receptors to bind ABA and interact with PP2Cs, allowing SnRK2s to be released from binding. Autophosphorylation activates the SnRK2s, allowing for transduction to take place. SnRK2s that have been activated include phosphorylate downstream substrate proteins, such as transcription factors. Abbreviations: Abscisic acid (ABA); dehydration-response element-binding protein/C-repeat binding factor (DREB/CBF); ethylene response 1 (ETR1); Arabidopsis NAC domain-containing protein (ANAC); mitogen-activated protein kinase (MAPK); sucrose nonfermenting-1 related kinase(SnRK); transcription factors (TFs); reactive oxygen species (ROS); protein phosphatase 2C (PP2C); ABA receptors (PYR/PYL/RCAR); ethylene response factors (ERFs); nitric oxide (NO)-ethylene (ET) interplay; ABA-responsive element-binding proteins/ABA-responsive element -binding factor (AREB/ABF); Etr, ethylene-resistant; Ctr, constitutive triple response; Ein, ethylene-insensitive; basic-domain leucine zipper (bZIP); dehydration-responsive elements/C-repeat (DRE/CRT); ABA-responsive elements (ABREs); potassium channel protein in *Arabidopsis thaliana* 1 (KAT1); slow anion channel 1 (SLAC1).

The plant response to abiotic stress involves cross-talk and interactions with various molecular pathways [140]. However, the responses to these abiotic conditions are dynamic and complex, and they can be reversible or irreversible [141,142]. Phytohormones play a critical role in regulating physiological processes and plant responses to abiotic stress. Hormones such as jasmonic acid (JA), abscisic acid (ABA), auxin, salicylic acid (SA), gibberellic acid (GA), and ethylene are essential for plant defence mechanisms [143]. These phytohormones activate signalling pathways that regulate downstream responses, mediating both growth and immunity against environmental stresses [144] (Figure 3). Among these hormones, ABA [145] and ethylene [146] play significant roles in abiotic stress, particularly in osmoregulation. ABA acts rapidly in signalling, at times independent of transcription factors. For example, ABA controls the stomatal aperture through mechanisms involving water transport and ion regulation [147]. Transcriptional regulation under salt-stress and water-deficit conditions has been studied in both ABA-dependent and ABA-independent mechanisms [148]. Water-deficit-induced cellular dehydration activates downstream signalling molecules, including metabolic enzymes and transcription factors, through increased endogenous ABA levels [148]. ABA-dependent mechanisms involve the expression of genes regulated by transcription factors like ABA-responsive element-binding proteins (AREBs) [148]. On the other hand, ABA-independent mechanisms involve the expression of transcription factors like dehydration-responsive element-binding proteins (DREB2) that regulate dehydration pathways [149]. In *Arabidopsis thaliana*, transcriptional control through the Abscisic acid-Insensitive 5 *(ABI5)* genes regulates plant response against various abiotic stresses [134]. Similarly, in *Xerophyta viscosa*, the regulatory expression of late embryogenesis abundant 4 *(LEA4)* genes plays a vital role in plant adaptation to stressful conditions [150]. The cascading activity of ABA signalling molecules enhances plant resistance against dehydration stress. In addition to ABA, SA also increases tolerance to water deficit in barley [151] and promotes drought resistance in other plant species [152].

Ethylene, along with ABA, regulates plant growth, survival, and defence against various environmental stresses [153]. The molecular response of ethylene to abiotic stress includes irradiation, drought, flooding, and temperature changes [154]. Ethylene-based responses depend on interactions with stress signals, metabolites, and other phytohormones [155]. The interplay between hormones like ABA and ethylene in drought response [154] has made the signalling pathways quite complex. Several membrane receptors, transcription factors, signalling protein kinases (PKs), and F-box proteins have been identified as signalling components in *A. thaliana* through bioinformatics and molecular genetics [156]. Among these, Ethylene Response 1 (ETR1) and Ethylene Sensor1 (ERS1) receptors, which possess conserved motifs of histidine kinases (HKs), play a crucial role in ethylene binding [157]. Constitutive Triple Response1 *(CTR1)*, encoding MAPK kinase (MKKK), is associated with receptor protein complexes and negatively regulates these proteins [158]. Another receptor factor, Ethylene Insensitive 3 (EIN3), activates numerous responsive genes, including Ethylene Response Factor 1 *(ERF1)*. This signalling cascade facilitates GCC binding, leading to the induction of ethylene-dependent secondary responses and the corresponding gene products that confer defence, growth, and survival in plants [159].

Primary metabolites such as sugars have been investigated for their active role in regulating plant metabolism and defence against abiotic stresses [12]. Soluble sugars, as signalling molecules, interact with ABA and ethylene to modulate plant growth and development. A study in grapevines demonstrated how interactive ABA and sugar signalling pathways control transport processes through enhanced expression of sugar transporter genes [160]. Lecourieux et al. [161] revealed increased expression of hexose transporters and protein kinase VVSK1 activity. Furthermore, sugar and light deficiency stimulate the activity of SnRK protein kinase [162]. Increased expression of SnRK2 protein kinase regulates plant metabolic activities and leads to higher sugar content in leaves [163]. Thus, sugar accumulation modulates plant metabolism and influences the accumulation of various secondary metabolites that are essential for adapting to environmental stresses.

## 7. Application of Plant Tissue Culture Techniques Associated with Plant Secondary Metabolites Production

While secondary metabolites can be obtained from plants grown under natural conditions, their production is influenced by environmental fluctuations [164]. Therefore, in vitro tissue culture serves as a crucial technique for enhancing the production of secondary metabolites [54]. In vitro culture for the production of these bioactive compounds offers a reliable resource and is not subject to quality fluctuations.

The synthesis of secondary metabolites through plant tissue culture is enhanced by identifying the optimal media composition, temperature, photoperiod, and plant growth regulators (PGRs) [165]. The use of suitable culture media, along with micronutrients, macronutrients, and plant hormones such as auxin, cytokinin, and gibberellins, plays a vital role in promoting the production of secondary metabolites [166] (Table 2).

Plant tissue culture involves the accumulation of biomass followed by the in vitro synthesis of secondary metabolites. Various approaches utilizing organized structures like callus, cell suspension, and shoot cultures have been employed for the synthesis of secondary metabolites [167].

**Table 2:** The influence of different growth regulators, as well as differential culture regimes on secondary metabolite accumulation in various plant species.

Plant Species	Medium + PGRs	Cultured Tisssue	Compound Name	Reference
*Camellia sinensis *L.	MS + 2,4-D + BAP	Callus	Catechin	[168]
*Arbutus andrachne *L.	WP + TDZ + NAA	Callus	Catechin	[169]
*Rauwolfia serpentina*	MS + Kn + BAP	Shoot	Phyllocladane diterpenoids	[170]
*Eurycoma longifolia*	MS + NAA +Kn	Cell suspension	Eurycomanone	[171]
*Talinum paniculatum*	MS + potassium nitrate	Hairy root	Saponin content	[172]
*Momordica charantia*	MS + sucrose	Hairy root	Flavonoids, phenolic acids	[173]
*Eleutherococcus koreanum*	½ MS + IBA + TDZ	Adventitious root	Eleutheroside B and E	[174]
*Eurycoma longifolia*	3/4 MS + IBA + NAA	Adventitious root	Flavonoids, phenolic content	[175]
*Astragalus membranaceus*	MS + IBA	Adventitious root	Saponin, flavonoid content	[176]
*Coleus blumei*	MMS + BA + NAA + sucrose	Callus and suspension	Rosmarinic acid	[177]
*Spilanthes acmella*	MS + BA + 2,4-D	Cell suspension	Scopoletin	[178]
*Ajuga bracteosa*	MS + BA + MeJ	Cell and callus	Monoterpene hydrocarbons	[179]
*Fagonia indica*	MS + TDZ	Callus	Gallic acid, quercetin	[180]
*Rosa damascena*	MS + BA + NAA	Callus	Tocopherols and β-carotene	[181]
*Salvia dolomitica*	MS + 2,4-D + Kn	Callus	α-Pinene, β-phellandrene	[182]
*Corylus avellana *L.	MS + BA + 2,4-D	Suspension	Taxol	[183]
*Linum usitatsimum *L.	MS + NAA	Callus	Lignans and neolignans	[184]
*Morus alba *L.	MS + Cefotaxime	Hairy root	Betulin and betulinic acid	[185]
*Solanum trilobatum *L.	MS + MeJ	Hairy root	Solasodine	[186]
*Salvia miltiorrhiza*	MS + MeJ + SA	Hairy root	Tanshinone	[187]
*Caralluma tuberculata*	MS + BA + 2,4-D	Callus	Phenolic and flavonoid content	[188]
*Rhodiola imbricata*	MS + BA + NAA	Callus	Phenylethanoids and phenylpropanoids	[189]
*Plumbago zeylanica *L.	MS + IBA	Root suspension	Plumbagin	[190]
*Verbena officinalis *L.	Schenk–Hildebrandt medium + 2,ip + TDZ	Shoot culture	Coumaran and hexadecenoic acid	[191]
*Thevetia peruviana*	Schenk–Hildebrandt medium + 2,4-D + Kn	Cell suspension	Phenolic compounds	[192]
*Oldenlandia umbellata *L.	MS + IBA + NAA	Adventitious root	Anthraquinones	[193]
*Vitis vinifera*	MS + IAA +GA_3_	Callus	Resveratol	[194]

MS—Murashige and Skoog, BA—6-Benzylaminopurine, IBA—indole-3-butyric acid, NAA—naphthalene acetic acid, 2,4-D—2,4-dichlorophenoxyacetic acid, GA_3_—gibberellic acid, TDZ—thidiazuron, Kn—kinetin, 2,iP—6-(γ,γ-Dimethylallylamino), MeJ—methyl jasmonate.

### 7.1. Callus and Cell Culture

The callus, which is an unorganized mass of cells, has proven to be highly efficient for the production of secondary metabolites due to its rapid growth and high reproducibility. In vitro callus culture has been successful in synthesizing various compounds such as anthocyanins, flavonoids, ajmaline alkaloids, α-tocopherol, serpentine, paclitaxel, scopolamine, and reserpine [195]. Glutathione and anthraquinones were produced through callus formation in *Nicotiana tabacum* and *Morinda citrifolia* [196]. Forskolin, a potent medicinal compound, has been found in calli obtained from the leaves of *Coleus forskohlii* [197]. Moreover, in vitro cell and callus cultures have been utilized to enhance the production of gymnemic acid in *Gymnema sylvestre* [198].

Elicitation by MJ (methyl jasmonate) was observed to be necessary for the enhanced synthesis of terpinolene and limonene monoterpenes under dark conditions in cell cultures of *Rosa damascene* [181]. Similarly, callus cultures of *Mentha piperita* have exhibited a higher accumulation of monoterpenes compared to naturally grown plants [199]. In Gingko biloba, cell suspension cultures recorded a higher accumulation of bilobalide and gingkolide [200]. Additionally, the combined effect of MJ and cyclodextrins in cell cultures of *Catharanthus roseus* has shown enhanced biosynthesis of alkaloids and terpenes [201].

### 7.2. Hairy Root Culture

Hairy root culture presents an alternative approach for enhancing biochemical properties and specific organic compounds, along with other classes of secondary metabolites [202]. This method involves transforming root cultures using *Agrobacterium rhizogenes*, resulting in the formation of hairy roots through the insertion of transfer DNA (T-DNA) [203]. The development of these hairy roots enables the accumulation of secondary metabolites in the newly grown plant roots [204].

Considerable attention has been given to optimize secondary metabolite production and selected plant species with favourable traits for hairy root (HR) induction. For instance, Barba-Espín et al. [205] demonstrated an increase in anthocyanin content in black carrot (*Daucus carota* ssp.) through HR induction by infecting it with an *R. rhizogenes* strain. Another example of specialized secondary metabolite production through hairy root culture involves introducing a gene of interest from *Hyoscyamus muticus* into *Atropa belladonna* using a binary vector system and *A. rhizogenes* [206]. Moreover, successful production of horhammericine alkaloids, tabersonine, and catharanthine compounds has been achieved from hairy root cultures of *Catharanthus roseus* transformed by the bacteria [207]. The potential of hairy root culture systems for optimal antiviral flavonoid production in *Isatis tinctoria* has also been demonstrated [208].

## 8. Conclusions and Future Prospects

Environmental factors have the ability to regulate the physiological and metabolic processes, consequently influencing the growth and productivity of plants. These factors, through abiotic stress, also impact the complex responses involved in the signal transduction of synthesized bioactive compounds. Therefore, the cascading signalling of stress factors has a profound effect on the regulation of phenotypic damage and metabolic functioning, which ultimately govern the plant’s inherent defence system.

The biosynthetic pathways responsible for producing secondary metabolites undergo modulation under different abiotic stresses and during various growing seasons, leading to either an increase or decrease in the content and accumulation of secondary metabolites. The adjustment of physiological and metabolic pathways under stress conditions is crucial for plants to adapt and develop an increased tolerance to such harsh environments. Studies have shown that specific secondary metabolites play roles as primary and regulator metabolites, intricately woven into plant metabolism. Researchers have endeavored to observe the dynamic and intricate plant responses to harsh conditions through cellular and molecular studies, but proteome analysis remains relatively unexplored.

To counter the influence of abiotic factors on the production of secondary metabolites, researchers have conducted studies involving a core group of genes to investigate the kinetics of plant responses under condition-dependent changes. Plant tissue cultures offer the advantage of synthesizing bioactive secondary metabolites regardless of temperature and other abiotic conditions. Therefore, there is substantial interest in large-scale production through in vitro cultures, necessitating comprehensive studies to explore the effects of environmental stimuli.

Investigations into the physiological and metabolic status of plants in response to stress stimuli could lay the foundation for integrating metabolic engineering and cell culture techniques in the synthesis of valuable plant secondary metabolites sustainably.

## Figures and Tables

**Table 1:** The effects of different environmental stresses on the content and accumulation of secondary metabolites in various plant species.

Plant Species	Abiotic Stress	Secondary Metabolite and Nature of Response	Reference
*Ocimum basilicum* (common basil)	AgNO_3_	The highest values of linalool and estragole compared with the control culture were obtained as 4.37 μg/g DW (25 μM treatment) and 3.30 μg/g DW (5 μM treatment), respectively.	[43]
*Chrysanthemum morifoilum* (garden mum)	Water	Anthocyanin content was observed to show an increasing trend by 1.71 and 3.5 times on days 7 and 5 of the stress period, respectively.	[44]
*Olea europaea* (common olive) cultivars	NaCl	Under 60 and 120 mM NaCl, the content of kaempferol in old leaves of Frantoio increased by three times (25.1 and 27.4 g g^−1^ FW, respectively). However, in Leccino, kaempferol remains unchanged at 60 mM NaCl and reduced 16 times (0.5 g g^−1^ FW) at 120 mM NaCl, when compared to control. Similarly, quercetin did not change up to 60 mM NaCl in both cultivars, while at the highest NaCl treatment (120 mM), it slightly increased in Frantoio.	[45]
*Lycopersicon esculentum * (garden tomato)	Pb	The treatment of Pb enhances the production of phenol, flavonoid, and anthocyanin content by 79.25%, 47.73%, and 58.25%, respectively, at 0.75 mM concentration.	[46]
*Artemisia annua* L. (sweet sagewort)	UV-B	UV-B-treated plants observed significant induction of 92 and 100% in total phenolic and flavonoid content after 3 h, respectively. However, artemisinin observed concentration up to 100%.	[47]
Citrus genotypes	Drought and heat stress	In Carrizo, impact of heat stress on secondary metabolite composition was 26.3%, whereas water stress had a lower contribution 18.8%. In Cleopatra, however, both heat and water stress influenced the secondary metabolite accumulation (21.1%), although the stress combination had a stronger contribution (26.2%).	[48]
*Genista tinctoria* (dyer’s greenweed)	UV radiation	The highest genistin content (3.03%) was demonstrated after 300 s of UV 254 nm treatment, followed by 2.06% accumulation after 120 s of UV 366 nm radiation. Similarly, 0.16–0.17% genistein content was observed after UV 254 nm treatment.	[49]
*Mentha pulegium* L. (pennyroyal)	Drought	The moderate and severe drought stress treatments increased TPC (17.2 and 30.3%, respectively) and TFC (35.9 and 33.7%, respectively).	[50]
*Salvia dolomitica* Codd (dolomite sage)	Drought	These total phenols and flavonoids were substantially reduced by moderate and severe drought stress compared with control (305.2, 53.2, and 20.5 mg GAE g^−1^, 105.7, 17.1, and 5.3 mg g^−1^, respectively).	[51]
*Brassica oleracea* L. convar. *acephala* (DC) Alef, var. *sabellica* L. (cabbage)	Drought	The content of proline and phytol in drought-stressed plants when compared to well-watered plants showed an increased trend of more than 22%, with values of 7.56 to 22.7 mg/plant for proline, respectively, and 22.1 to 35.6 mg/plant for phytol, respectively.	[52]
Cotton genotypes	Drought and Salinity	In Zhongmian 23, TPC increased significantly under drought and/or salinity compared to their controls, while under salinity, it remained unaffected at 10% SMC. However, in Zhongmian 41, TPC was significantly increased under D + S relative to control and remained unchanged under drought, but it was significantly decreased under salinity when compared to control. Both drought and/or salinity stress increased TFC content. However, under salinity, it decreased in Zhongmian 23 and increased in Zhongmian 41 at 10% SMC, while at 4% SMC it remained unaffected compared to control.	[53]
*Carthamus tinctorius* L. (safflower)	Salinity	Significant increase of 34% in TPC, 13% in TFC, and 12% in TFL was observed in salinity-stressed plants when compared to nonstressed plants.	[54]
*Solanum nigrum *L. (black nightshade)	NaCl	Significant increases were observed in both lutein and β-carotene at 100 mM NaCl, while at 50 and 150 mM NaCl, both compounds showed reduced accumulation. Quercetin levels increased 2.6-fold compared to quercetin 3-β-D-glucoside at 0 and 50 mM NaCl treatments, whereas they were the same for the 100 mM treatment and 2-fold lower at 150 mM NaCl treatment.	[55]
*Camellia japonica* cultivars (Japanese camellia)	Temp.	In ‘Jiangxue’, the content of palmitic acid, stearic acid, and oleic acid gradually decreased during cold treament, while α-linolenic acid increased significantly. Similar tendencies were found in ‘Desire’ and ‘Nuccio’s Bella Rossa’, but the changes were not as significant as in ‘Jiangxue’.	[56]
*Musa* spp. (banana)	Temp.	The TPC of Simili radjah increased 2.9-fold, from 6.3 (26 °C) to 18.5 ng/g GAE (20 °C), whereas it increased 4.8-fold from 8.6 (26 °C) to 41.6 ng/g GAE (20 °C) for Dole.	[57]
*Scutellaria lateriflora* (mad dog skullcap)	Light	The accumulation of baicalin was promoted by blue light, 0.96–2.00 times higher than under white light. Flavonoids showed 0.93–1.54 times accumulation in blue light than under white light. However, 3,4-dihydroxyphenylacetic acid observed the highest concentration of (33.56 mg 100 g^−1^ DW) in the presence of white light.	[58]
*Silybum marianum* L. (milk thistle)	Light	Under red light, silymarin content (18.67 mg/g DW) was almost double compared to control (9.17 mg/g DW). Conversely, taxifolin accumulation (0.480 mg/g DW) was found to be maximum under continuous white light, which was almost eightfold higher than control (0.063 mg/g DW).	[59]
*Rhododendron tomentosum* (marsh Labrador tea)	Cu and Ni	About a twofold increase in pcymene and sabinene was observed in Cu- and Ni-treated plants compared with control. Pcymene and sabinene contents were about 23.0% and 17.7% vs. 6.9% (control), respectively; and 5.4% and 3.0% vs. 1.7% (control), respectively. On the other hand, δ-cadinene was decreased by 1.0% and 1.1% vs. 4.1% (control), respectively, in the total level of sesquiterpene hydrocarbons.	[60]
*Corylus avellana *L. (common hazel)	Al	Extracellular taxol content showed an upward trend at 100 µM of Al treatment and was 38-fold higher than that of the control medium. However, cell-associated taxol content at 50 and 100 µM of Al concentration was enhanced by 11.4- and 8.3-fold, respectively, compared to control cells.	[61]
*Linum usitatissimum *L. (flax)	UV + photoperiod	Under UV + dark and UV + photoperiod, 1.12- and 2.82-fold enhancement in TPP was noted, respectively, in response to 3.6 kJ/m^2^ of UV-C radiations. However, at similar conditions, TFP showed 1.42- and 2.94-fold enhancement compared to their respective controls.	[62]
*Duboisia* *Species* (corkwood)	Light and temperature	Scopolamineproduction showed a negative trend by increased light intensity up to 350 μmol/m^2^ × s, light exposure up to 24 h/d, and temperature (28 °C).	[63]

TPC—total phenolic content, TFC—total flavonoid content, TFP—total flavonoid production, TPP—total phenolic production, SMC—soil moisture content, TFL—total flavonols, DW—dry weight, FW—fresh weight, D—drought, S—salinity.
